# Analysis of Nuclear Export Sequence Regions of FUS-Related RNA-Binding Proteins in Essential Tremor

**DOI:** 10.1371/journal.pone.0111989

**Published:** 2014-11-06

**Authors:** Oswaldo Lorenzo-Betancor, Kotaro Ogaki, Alexandra Soto-Ortolaza, Catherine Labbé, Carles Vilariño-Güell, Alex Rajput, Ali H. Rajput, Pau Pastor, Sara Ortega, Elena Lorenzo, Audrey J. Strongosky, Jay A. van Gerpen, Ryan J. Uitti, Zbigniew K. Wszolek, Owen A. Ross

**Affiliations:** 1 Department of Neuroscience, Mayo Clinic, Jacksonville, Florida, United States of America; 2 Djavad Mowafaghian Center for Brain Heath, Department of Medical Genetics, University of British Columbia, Vancouver, Canada; 3 Division of Neurology, University of Saskatchewan and Saskatoon Health Region, Saskatoon, Canada; 4 Neurogenetics Laboratory, Division of Neurosciences, Center for Applied Medical Research, University of Navarra School of Medicine, Pamplona, Spain; 5 Centro de Investigación Biomédica en Red de Enfermedades Neurodegenerativas, CIBERNED, Instituto de Salud Carlos III, Madrid, Spain; 6 Department of Neurology, Hospital Universitari Mutua de Terrassa, Terrassa, Barcelona, Spain; 7 Department of Neurology, Mayo Clinic, Jacksonville, Florida, United States of America; 8 Mayo Graduate School, Neurobiology of Disease, Mayo Clinic, Jacksonville, Florida, United States of America; International Centre for Genetic Engineering and Biotechnology, Italy

## Abstract

**Background and Objective:**

Genes encoding RNA-binding proteins, including FUS and TDP43, play a central role in different neurodegenerative diseases such as amyotrophic lateral sclerosis and frontotemporal lobar degeneration. Recently, a mutation located in the nuclear export signal (NES) of the *FUS* gene has been reported to cause an autosomal dominant form of familial Essential tremor.

**Material and Methods:**

We sequenced the exons coding the NES domains of five RNA-binding proteins (*TARDBP*, *hnRNPA2B1*, *hnRNPA1*, *TAF15* and *EWSR1*) that have been previously implicated in neurodegeneration in a series of 257 essential tremor (ET) cases and 376 healthy controls. We genotyped 404 additional ET subjects and 510 healthy controls to assess the frequency of the EWSR1 p.R471C substitution.

**Results:**

We identified a rare EWSR1 p.R471C substitution, which is highly conserved, in a single subject with familial ET. The pathogenicity of this substitution remains equivocal, as DNA samples from relatives were not available and the genotyping of 404 additional ET subjects did not reveal any further carriers. No other variants were observed with significant allele frequency differences compared to controls in the NES coding regions.

**Conclusions:**

The present study demonstrates that the NES domains of RNA-binding proteins are highly conserved. The role of the EWSR1 p.R471C substitution needs to be further evaluated in future studies.

## Introduction

**Figure 1:**
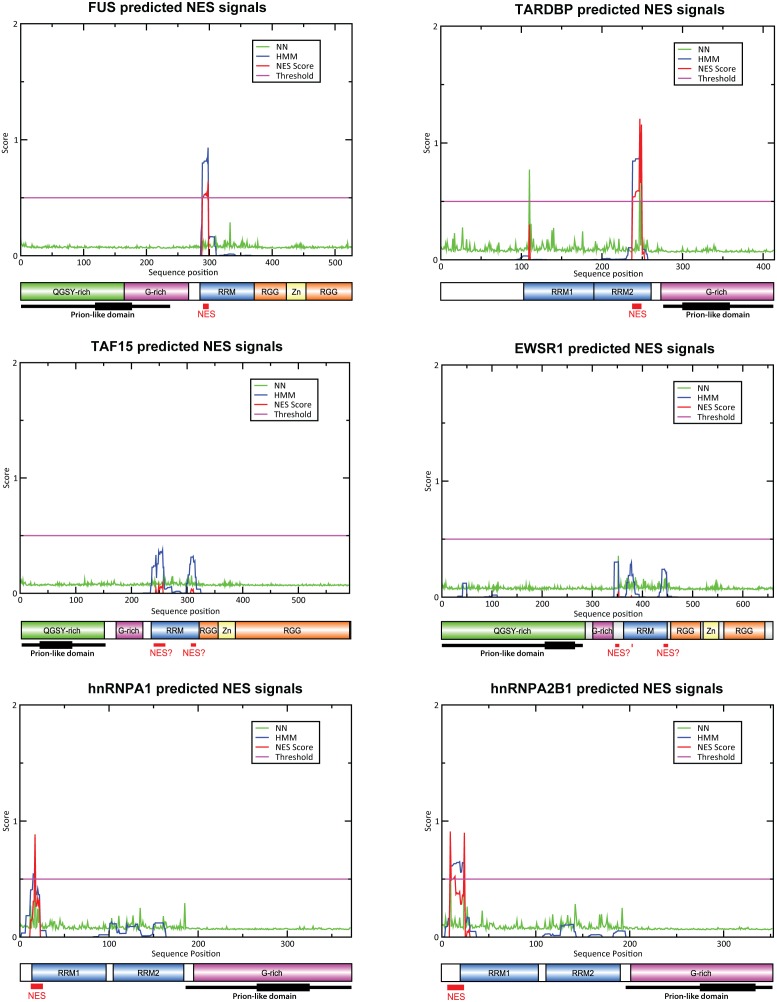
Nuclear export signal (NES) prediction of candidate proteins based on the NetNES 1.1 prediction tool [16]. NN = neural network algorithm; HMM = hidden Markov Model algorithm; NES score = combination of NN and HMM algorithms; QGSY-rich = glutamine, glycine, serine, tyrosine rich region; G-rich = glycine rich region; RRM = RNA recognition motif; RGG = Arg-Gly-Gly rich domain; Zn = zinc finger domain; The ? denotes that the NES predicted location does not surpass the NetNES established threshold. The thin black line denotes the prion-like domain location and the thick black line represents the highest score core region according to the Alberti algorithm [20].

Essential tremor (ET) is the most common movement disorder of the elderly and is characterized by a postural or motion tremor [1]. Recently, exome sequencing in a large pedigree with an autosomal dominant form of familial ET proposed a rare mutation in the nuclear exporting signal region (NES; p.Q290X) of *Fused in Sarcoma* gene (*FUS*; OMIM*137070) as pathogenic [2]–[4]. FUS is a RNA-binding protein carrying a canonical RNA recognition motif (RRM), an NES and a putative prion-like domain (Figure 1).

Several RNA-binding proteins harboring these domains have been implicated in the development of different neurodegenerative diseases. For example, mutations in TAR DNA binding protein (*TARDBP*; OMIM*605078) and *FUS* cause familial amyotrophic lateral sclerosis (ALS) [5]–[10]. Moreover, mutations in *heterogeneous nuclear ribonucleoprotein A1* (*hnRNPA1*; OMIM*164017) and *A2/B1* (*hnRNPA2B1*; OMIM*600124) have been described in families with multisystem proteinopathy and ALS [11], *TATA box-binding protein-associated factor 2N* (*TAF15*; OMIM*601574) and *Ewing sarcoma breakpoint region 1* (*EWRS1*; OMIM*133450) have been implicated both in ALS and frontotemporal lobar degeneration with ubiquitin-positive inclusions (FTDL-U) [12]–[14].

Our previous sequencing studies of the entire coding region of *FUS* gene in ET did not identify any additional pathogenic variants within the NES domain [3], [4]. In the present study we have sequenced the predicted NES locations of these additional RNA-binding proteins in a cohort of ET subjects and controls, in order to identify novel mutations in those regions that may be responsible for disease.

## Materials and Methods

All individuals gave their written informed consent and the study was approved by the Mayo Clinic Institutional Review Board, Jacksonville, Florida and the respective local Ethical Committees.

**Figure 2:**
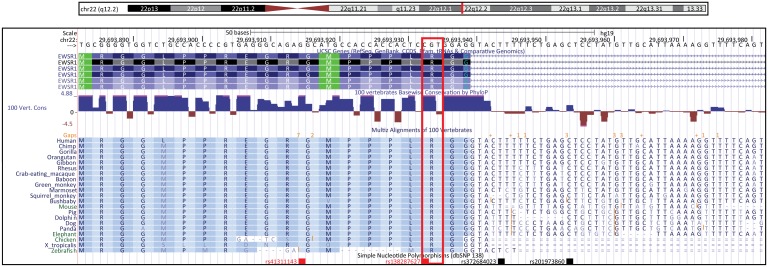
Conservation of EWSR1 p.R471 amino acid. Species alignment of EWSR1 p.R471 amino acid showing its highly preservation across evolution and the exact location of p.R471C substitution (rs138287627). Picture extracted from the UCSC Genome Browser (http://genome.ucsc.edu/cgi-bin/hgGateway).

The Mayo Clinic ET series includes 257 patients, 151 individuals with familial ET and 106 sporadic ET subjects (Table 1). A diagnosis of ET was established according to the Consensus Statement of the Movement Disorder Society on Tremor [15] by an experienced neurologist specialized on movement disorders (JAvG, RJU and ZKW). A series of 376 healthy subjects from Mayo Clinic, Jacksonville, was sequenced to establish the minor allele frequency (MAF) of identified mutations in a control population. All participants in the study are unrelated, non-Hispanic Caucasians recruited at Mayo Clinic, Jacksonville. In order to replicate the results of our study, we further genotyped two additional ET series and matched healthy controls to establish the frequency of the EWSR1 p.R471C substitution which is located in a highly conserved region of the protein (Figure 2). We genotyped 291 Canadian ET patients and 328 matched healthy subjects and 113 Spanish ET patients and 182 matched healthy subjects (Table 1).

**Table 1:** Demographic data of Discovery and Replication Samples.

	Discovery Sample				Replication Samples								Whole Sample			
	USA				Replication Sample 1 (Canada)				Replication Sample 2 (Spain)							
	Fam ET	Spo ET	All ET	Cont	Fam ET	Spo ET	All ET	Cont	Fam ET	Spo ET	All ET	Cont	Fam ET	Spo ET	All ET	Cont
	(n = 151)	(n = 106)	(n = 257)	(n = 376)	(n = 113)	(n = 178)	(n = 291)	(n = 328)	(n = 39)	(n = 74)	(n = 113)	(n = 182)	(n = 303)	(n = 358)	(n = 661)	(n = 886)
Age*(SD)	74.28(11.66)	75.77(12.02)	74.90(11.83)	67.12(12.26)	71.76(15.08)	74.91(12.93)	73.65(13.92)	73.26(12.37)	60.00(15.10)	65.96(14.55)	63.88(15.01)	68.92(10.43)	71.54(14.20)	73.30(13.56)	72.48(13.89)	69.63(12.25)
Age*range	35–96	46–98	35–98	29–88	32–99	23–97	23–99	27–99	19–88	22–91	19–91	12–110	19–99	22–98	19–99	12–110
AAO§(SD)	47.04(19.91)	56.10(18.73)	50.66(19.95)	NA	52.10(19.06)	56.33(16.39)	54.74(17.56)	NA	49.77(18.83)	55.85(20.87)	53.86(20.43)	NA	49.21(19.61)	56.16(18.12)	53.00(19.12	NA
AAO§range	5–84	6–88	5–88	NA	15–87	12–87	12–87	NA	14–78	10–89	10–89	NA	5–87	6–89	5–89	NA
Female,%	54.31	51.89	53.31	55.32	58.41	55.62	56.70	69.21	53.85	50.00	51.33	54.95	55.78	53.35	54.46	60.38

Fam ET = familial essential tremor; Spo ET = sporadic essential tremor; Cont = Healthy controls; y = years; AAO = age at onset; SD = standard deviation; NA = data not applicable.

*Age was not available for 57 subjects (6 familial, 17 sporadic cases and 34 controls) from Replication Sample 1 and for 11 subjects (1 familial, 3 sporadic cases and 7 controls) from Replication Sample 2.

§ AAO was not available for 22 subjects (10 familial and 12 sporadic cases) from the Discovery Sample, for 27 subjects (14 familial and 13 sporadic cases) from the Replication Sample 1 and for 6 subjects (4 familial and 2 sporadic cases) from Replication Sample 2.

The NetNES prediction server (http://www.cbs.dtu.dk/services/NetNES/) [16] was used to identify potential NES signals in the candidate genes. This online tool works with amino acid sequences and combines both neural networks (NN) and hidden Markov models (hMM) in its prediction algorithm. The integration of both models allows us to combine the superior observed specificity of the hMM with the observed superior sensitivity of the NN [16]. Once the NES amino acid signals were identified (Figure 1), we performed bidirectional sequencing of the exons of each gene coding for these specific regions (see Table S1 for specific primers).

Variants were numbered according to standard nomenclature based on RefSeq mRNA and peptide accession numbers for each gene (*TARDBP*: NM_007375.3, NP_031401.1; *hnRNPA1*: NM_002136.2, NP_002127.1; *hnRNPA2B1*: NM_031243.2, NP_112533.1; *TAF15*: NM_139215.2, NP_631961.1; *EWSR1*: NM_005243.3, NP_005234.1). PolyPhen-2 (http://genetics.bwh.harvard.edu/pph2/index.shtml) and SIFT (http://sift.bii.a-star.edu.sg/) algorithms were used to assess the impact of the amino acid substitutions on the protein structure. The virtual effect of intronic variants on splicing was assessed using the Human Splicing Finder (HSF) algorithm (http://www.umd.be/SSF/) [17]. Allelic association analysis and Bonferroni correction for multiple testing were performed with PLINK v.1.07 software (http://pngu.mgh.harvard.edu/purcell/plink/) [18].

## Results

Our sequencing analysis of 257 patients with ET did not identify any novel variants in the predicted NES coding regions of the candidate genes. We identified two previously described missense variants in the *EWSR1* gene (rs41311143, p.G465S and rs138287627, p.R471C; Table 2), which are predicted to be probably damaging and benign by the Polyphen-2 algorithm, respectively. The minor allele frequency (MAF) of the EWSR1 p.G465S variant was the same in ET cases and controls (MAF 1.5%) whereas the p.R471C substitution was observed in only one patient with autosomal dominant familial ET. Unfortunately, DNA from the rest of the family members was not available to asses for co-segregation of this variant with disease. The genotyping of 404 additional ET patients and 510 healthy controls did not reveal any additional EWSR1 p.R471C carriers.

**Table 2:** Genotype counts of variants identified in predicted NES regions of candidate genes from the Discovery Sample.

					ET Patients (n = 257)				Controls (n = 376)								
Gene	Location	rs number	Mutation	AA change	Major	Het	Minor	MAF	Major	Het	Minor	MAF	EVS MAF	1 kG MAF	P value^ a^	PolyPhen-2	SIFT score
EWSR1	Int 10	rs41309649	c.1046–17C>G	Intronic	250	7	0	0.014	366	8	0	0.010	0.01	0.01	0.63	NA	NA
EWSR1	Int 11	rs3761426	c.1164+37T>G	Intronic	186	63	8	0.154	263	90	21	0.120	0.12	0.15	0.29	NA	NA
EWSR1	Ex 13	rs41311143	c.1393G>A	p.G465S	250	7	0	0.014	358	11	0	0.015	0.01	0.01	0.85	Probably damaging (0.96)	Tolerated (0.19)
EWSR1	Ex 13	rs138287627	c.1411C>T	p.R471C	256	1	0	0.002	369	0	0	0	7.68×10^−5^	NA	0.23	Benign (0.013)	Tolerated (0.07)
EWSR1	Int 13	rs3747142	c.1417+51A>G	Intronic	184	68	5	0.152	258	103	8	0.137	0.12	0.14	0.65	NA	NA
EWSR1	Int 13	NA	c.1417+68_1417+71delGATT	Intronic	252	3	0	0.006	361	8	0	0.011	NA	NA	0.36	NA	NA
hnRNPA1	Int 1	rs2071391	c.16–57G>A	Intronic	186	59	7	0.145	265	103	8	0.137	NA	0.24	0.51	NA	NA
hnRNPA2B1	Int 2	NA	c.42+17T>C	Intronic	256	1	0	0.002	375	0	0	0	NA	NA	0.23	NA	NA
hnRNPA2B1	Int 3	rs41275982	c.153+4T>C	Intronic	242	15	0	0.029	355	19	1	0.025	0.03	0.03	0.90	NA	NA
TAF15	Int 12	rs4251774	c.1006+34A>G	Intronic	247	10	0	0.019	373	3	0	0.004	0.01	0.01	0.01	NA	NA
TARDBP	Ex 6	rs147795017	c.1122T>C	p.Y374Y	255	1	0	0.002	365	0	0	0	7.7×10^−5^	NA	0.23	NA	Tolerated (1)

Genes are sorted in alphabetical order. ET = essential tremor; AA = Amino acid; EVS = Exome Variant Server; 1 kG = 1000 Genomes; MAF = minor allele frequency; Ex = exon; Int = intron; Het = count of heterozygous carriers; NA = not applicable; SIFT = Sorting Tolerant From Intolerant algorithm.

a Uncorrected p-value calculated with PLINK case-control association analysis.

Across the candidate genes we observed a known synonymous variant in the *TARDBP* gene (rs147795017, p.Y374Y; Table 2) and eight intronic variants, two of which were novel (*hnRNPA2B1* c.42+17T>C and *EWSR1* c.1417+68_1417+71delGATT; Table 2). The *hnRNPA2B1* c.42+17T>C mutation was present in a single sporadic case and was absent in a series of 376 healthy controls. The HSF algorithm predicts this mutation to change the exon 2 splicing site including 15 intronic nucleotides between exons two and three, which would cause an in-frame insertion of five amino acids (VLCQQ), but RNA from the mutation carrier was not available for examination. The *EWSR1* c.1417+68_1417+71delGATT variant was present in three ET subjects, two familial ET cases and one sporadic subject (MAF = 0.006), but was also present in 8 out of 376 healthy controls (MAF = 0.011) excluding a possible role in ET pathology. The frequency of *TAF15* c.1006+34A>G intronic variant was significantly different between cases and controls (p = 0.007), however this level of significance would not remain after Bonferroni correction for multiple testing.

## Discussion

The involvement of mutated RNA-binding proteins in several neurodegenerative disorders suggests that this family of proteins may be relevant across heterogeneous disease phenotypes. The identification of a nonsense mutation in the NES domain of the FUS protein (p.Q290X) in a large kindred with autosomal dominant ET has raised interest in the role of these genes in this common movement disorder. In the present study we screened the predicted NES regions of other RNA-binding proteins that have been associated with neurodegeneration but did not identify any novel variants related to the ET phenotype.

*FUS* mutations have been proposed to be involved both in ALS [5], [7] and in ET [2]. However, the described mutations in both diseases are located in different domains of the protein. While the ALS mutations affect the RGG domain [7], the mutation causing ET results in a premature stop codon located in the NES region of the protein [2]. Additionally, functional analyses have shown that the pathogenic effect of the ET-specific *FUS* mutation, whose mRNA is degraded by the nonsense-mediated decay (NMD) pathway, differ from those of the ALS mutations, whose mRNAs do not undergo this kind of degradation [2]. This fact suggests that the affected domain of the protein and type of mutation plays a critical role in determining the disease phenotype developed by mutation carriers.

A recent study has shown that ∼1% of human protein-coding genes contain a potential prion-like domain and of this 1%, there is a 12-fold enrichment from proteins containing also a canonical RRM [19]. Thus, ∼11.7% of human protein-coding genes containing a candidate prion domain also harbors an RRM. The high percentage of RRM proteins among prion-like candidates suggests that human RNA-binding proteins could have a greater trend towards aggregation and therefore play a role in neurodegeneration [19]. Therefore, although we sequenced only the NES domain of these candidate genes, further investigation of other domains such as the RRM may be warranted. However, whether ET is a neurodegenerative disorder remains controversial and there is no clear evidence of protein aggregation in the disease pathology. Thus, further understanding of the genetic determinants underlying ET risk will hopefully clarify the pathogenic processes and provide a clearer picture of disease etiology.

Although we could not establish the cosegregation of the EWSR1 p.R471C substitution in the index family due to the lack of DNA samples from affected relatives, nor its presence in other ET populations, the EWSR1 p.R471C substitution is a candidate variant that needs to be further screened in future ET studies.

## Supporting Information

Table S1 **Sequencing primers.**
(DOCX)
