# Supplementary material for: Analysis of Nuclear Export Sequence Regions of FUS-Related RNA-Binding Proteins in Essential Tremor
Source: PLoS One. 2014 Nov 6;9(11):e111989. doi: 10.1371/journal.pone.0111989 (PMC4222957; doi:10.1371/journal.pone.0111989)
Supplement: Table S1 — Sequencing primers. (DOCX) [file pone.0111989.s001.docx]

**Table S1.** Sequencing primers.

| **Gene (exon)** | **FOR** | **REV** |
| --- | --- | --- |
| **TARDBP** | | |
| Exon 6 | GCTTATTTTTCCTCTGGCTTT | AAAAATTTGAATTCCCACCA |
| **hnRNPA2B1** | | |
| Exon 2-3 | CCTTTGATAATCACGGTCTAC | AGGATCCCTCATTACCTTTC |
| **hnRNPA1** | | |
| Exon 2 | GACCTGAACGAACAATAAGTG | TTTGCCAACAAAGCTCTAAC |
| **TAF15** | | |
| Exon 10 | GAACAGAATGTCATAACCAAGG | TTTAAGAGACAAGGCGTACTG |
| Exon 12 | TGAGGACATGTCAGTTACTCC | AAAGCAGTAGCAAGTTCACAC |
| **EWSR1** | | |
| Exon 10-11 | TTGTAAGGTTTGTAGCTTGC | GGCAACCTTAGAAAACAACTC |
| Exon 13 | AGAGAAGATTACAGGCAGACC | ACATTCATTGTTAGCTCTCG |
